# Combined intra- and extracapsular stabilization for CCL rupture in a toy-breed dog: a case report

**DOI:** 10.3389/fvets.2025.1692055

**Published:** 2025-11-27

**Authors:** Sung Su Park, Ho Jae Han

**Affiliations:** 1 IU Animal Medical Center, Seongnam, Gyeonggi Province, Republic of Korea; 2 Department of Veterinary Physiology, College of Veterinary Medicine, Research Institute for Veterinary Science, Seoul National University, Seoul, Republic of Korea

**Keywords:** canine (dog), cranial cruciate ligament (CrCL) rupture, intra articular, extra articular, toy-breed dog

## Abstract

Cranial cruciate ligament (CrCL) rupture is a frequent orthopedic condition leading to hindlimb dysfunction in dogs. While tibial plateau leveling osteotomy (TPLO) has become the predominant surgical choice in large breeds, its use in toy-breed dogs remains controversial due to anatomical constraints and implant limitations. This case report presents a 10-year-old, 2.0 kg Maltese with CrCL rupture and underlying cardiac disease, for whom osteotomy was contraindicated. A hybrid stabilization technique was performed, combining intra-articular suture placement with extracapsular anchoring via bone tunnels, intentionally avoiding use of the lateral fabella. The procedure resulted in rapid postoperative limb function recovery and no observed complications. This outcome highlights the potential clinical value of non-osteotomy-based stabilization in ultra-small dogs, particularly in cases where traditional methods pose excessive risk. Continued case accumulation and long-term monitoring are necessary to further validate the safety and efficacy of this approach.

## Introduction

Cranial cruciate ligament (CrCL) injury remains one of the most common causes of lameness in canine patients. The biomechanical consequences of CrCL rupture include instability of the stifle joint, meniscal trauma, and progressive degenerative changes over time (1, 2).

To treat this condition, a variety of surgical interventions have been developed, typically classified into extracapsular, intracapsular, and osteotomy-based techniques. Among these, tibial plateau leveling osteotomy (TPLO) has gained widespread acceptance, particularly in larger dogs, due to its ability to neutralize cranial tibial thrust and restore joint function (3, 4).

However, when applied to toy-breed dogs, TPLO and other osteotomy-based procedures, such as tibial tuberosity advancement (TTA) and cranial tibial wedge osteotomy (CTWO), often encounter limitations. These include reduced cortical bone thickness, small implant accommodation, and a heightened risk of intraoperative or postoperative complications (5, 6). In addition, these techniques predominantly target translational instability, often failing to sufficiently control internal rotational forces, which may be especially relevant in small dogs (7).

Previous studies have generally reported that TPLO and TTA yield acceptable complication rates and functional outcomes even in small-breed dogs (8). Nevertheless, in ultra-small or toy-breed patients weighing less than approximately 5 kg, several anatomical and biomechanical factors warrant a more conservative application of osteotomy-based procedures. The residual tibial tuberosity after osteotomy is often extremely thin, predisposing it to intraoperative or delayed fracture (9), and the use of rigid fixation plates may induce localized stress-shielding or implant-associated bone density reduction over time (10). Such mechanical and biological considerations suggest that, in very small dogs, non-osteotomy alternatives—including intra-articular, extracapsular, or combined stabilization techniques—may provide sufficient stability while minimizing the structural risks associated with osteotomy.

As an alternative, non-osteotomy stabilization methods have been proposed, including modifications that combine intra-articular and extracapsular elements to improve joint control (11, 12). Although individual case reports inherently provide limited statistical strength, they can serve as preliminary investigations that highlight novel surgical concepts and generate hypotheses for future clinical validation. The present report therefore explores the feasibility and short-term clinical outcome of a combined intra- and extracapsular stabilization technique as a non-osteotomy alternative for cranial cruciate ligament rupture in ultra-small dogs, providing early insight into its biomechanical rationale and potential clinical relevance.

This case specifically involves a geriatric toy-breed dog with complex comorbidities, in which a combined stabilization strategy was utilized to avoid osteotomy. The report discusses the rationale, surgical technique, and outcome, with emphasis on its potential applicability in similar high-risk toy breed dog.

## Case description

A 10-year-old castrated male Maltese, weighing 2.0 kg, was referred for evaluation of acute, non-weight-bearing lameness in the right hind limb. One year prior, the patient had undergone bilateral tibial tuberosity transposition (TTT) for correction of medial patellar luxation. Medical history also included stage B2 myxomatous mitral valve disease managed. The patient was receiving pimobendan (Vetmedin^®^, Boehringer Ingelheim, Germany; 0.25 mg/kg PO BID), a calcium sensitizer and phosphodiesterase III inhibitor that enhances myocardial contractility and induces balanced vasodilation. Furosemide (Lasix^®^, Handok Pharmaceutical, Korea; 0.5 mg/kg PO BID), a loop diuretic acting on the Na^+^–K^+^−2Cl^−^ symporter of the thick ascending limb of the loop of Henle, was used to relieve pulmonary congestion. It's natriuretic and kaliuretic effects warranted periodic electrolyte monitoring for potential hypokalemia. Enalapril (Enap^®^, Donga ST, Korea; 0.5 mg/kg PO BID), an angiotensin-converting enzyme inhibitor, was given to reduce afterload and suppress renin-angiotensin activation.

**Figure 1:**
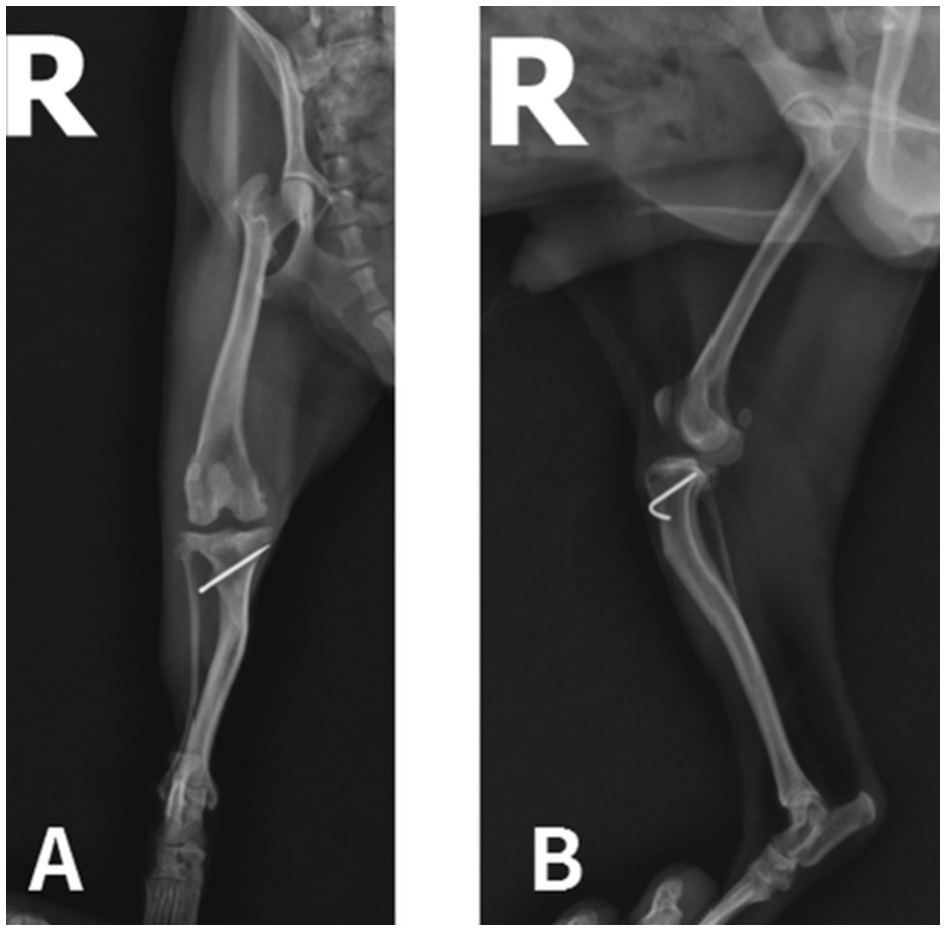
Postoperative radiographs from previous medial patellar luxation surgery. **(A)** Ventrodorsal view showing prior TTT fixation. **(B)** Lateral view revealing CDM, indicating CrCL rupture.

On orthopedic examination, the right stifle demonstrated a positive cranial drawer motion (CDM). Radiographic assessment confirmed cranial displacement of the tibia and mild periarticular osteophyte formation, consistent with CrCL rupture. Structural alterations from the previous TTT procedure, including cortical irregularities and tibial morphology changes, rendered the site unsuitable for osteotomy-based stabilization. Given the patient's cardiac comorbidity and low body weight, a less invasive approach was considered suitable (Figure 1).

Pre-anesthetic screening, including thoracic radiographs, complete blood count, and serum biochemistry, revealed no contraindications to anesthesia. Premedication consisted of butorphanol (0.3 mg/kg) and midazolam (0.2 mg/kg), followed by induction with propofol (6 mg/kg) and maintenance with sevoflurane. Regional analgesia was provided via local infiltration of bupivacaine, and postoperative pain management included a 72-h transdermal fentanyl patch (12 μg/patch).

**Figure 2:**
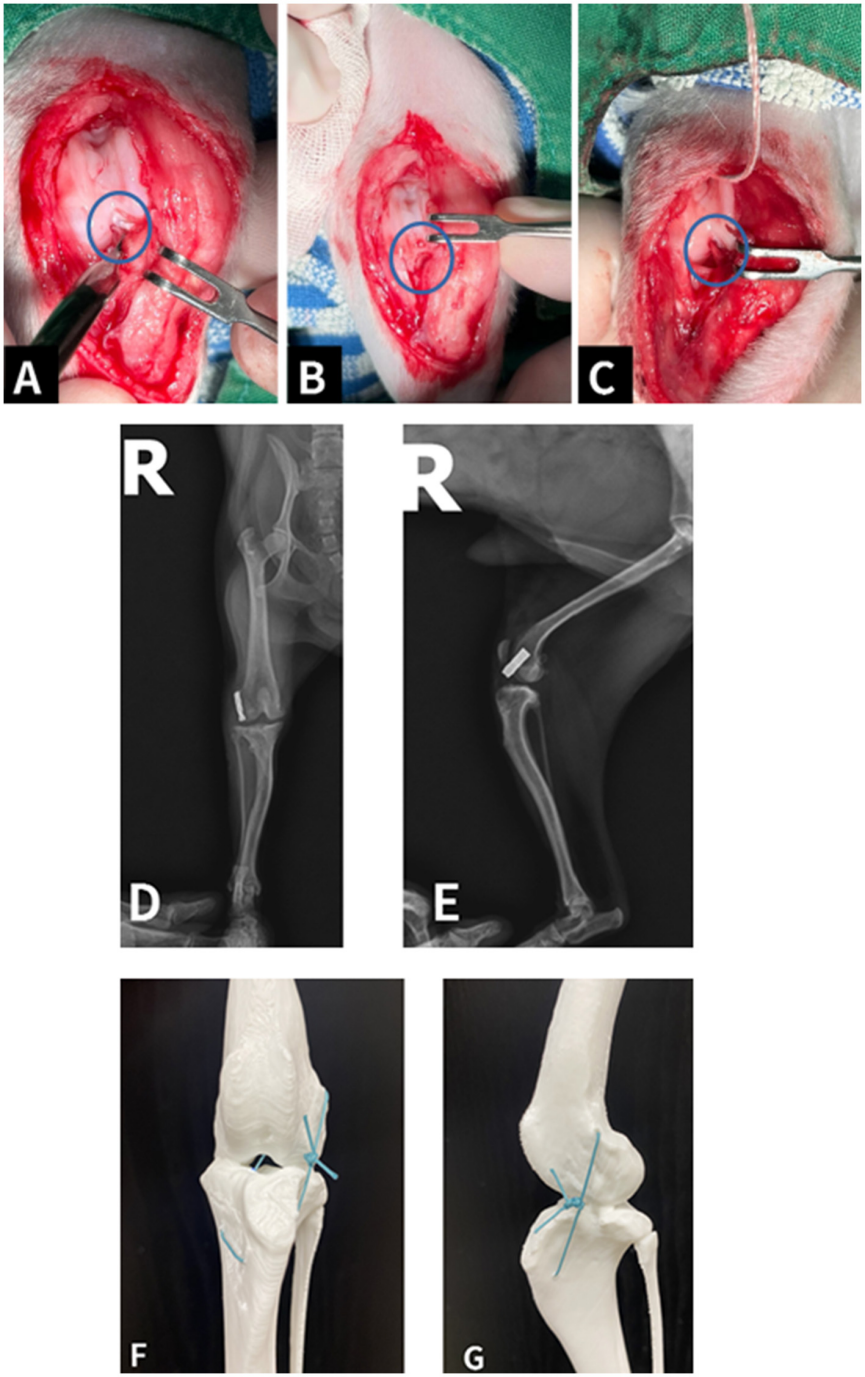
Intraoperative photographs. **(A)** Ruptured cranial cruciate ligament (blue circle) observed within the stifle joint. **(B)** Appearance of the joint after debridement of the ruptured ligament (blue circle). **(C)** Final placement of the intra-articular prosthetic ligament, mimicking the anatomical position of the native CrCL (blue circle). Postoperative radiographs following the combined intra- and extracapsular stabilization procedure. **(D)** Ventrodorsal view demonstrating lateral crimp fixation of the prosthetic ligament. **(E)** Lateral view confirming elimination of CDM and appropriate tibial alignment without displacement. Three-dimensional printed bone models illustrating the trajectory of the Polyethylene filament line. **(F)** Frontal view showing the intra-articular ligament passing from the distal lateral femur to the medial tibia. **(G)** Lateral view showing the extracapsular extension and fixation on the lateral aspect of the tibia, resembling conventional suture anchor techniques. For demonstration purposes, a simple knot was used in the model in place of the actual stainless-steel crimp.

Surgical stabilization involved a hybrid technique integrating intra-articular and extracapsular components. Bone tunnels were created at the anatomical attachment sites of the ruptured cranial cruciate ligament using a 1.1-mm drill bit, reproducing the native orientation of the CCL. A synthetic ligament (Ligafiba 150, Veterinary Instrumentation, UK) and the corresponding clamp system were applied through these tunnels. The limb was maintained in extension during fixation, and tension was adjusted to eliminate cranial drawer motion without inducing excessive tibial rotation. Fixation was secured using the Ligafiba clamp in a modified fabellotibial configuration, bypassing the use of the lateral fabella (Figure 2).

Postoperative recovery was monitored primarily based on the initiation of weight-bearing ambulation rather than a numerical lameness or pain score, as the patient's small body size and compliant behavior allowed for clear functional observation. The patient's compliance and ultra-small size allowed for functional observation that served as a highly reliable, patient-specific analog to a formal lameness score. A standard Robert Jones bandage was applied for 7 days postoperatively to provide soft-tissue support and reduce local swelling. After bandage removal, strict cage rest was maintained for approximately 4 weeks to minimize excessive motion and promote stable bone tunnel healing. No active physiotherapy was initiated during this period; instead, the patient was encouraged to perform limited voluntary movements within the cage to facilitate gentle, natural rehabilitation. After 1 month, controlled leash walking was introduced to gradually restore limb function. Given the minimally invasive nature of the procedure and the absence of osteotomy, recovery progressed uneventfully, and satisfactory functional stability was achieved through restricted activity alone.

Follow-up evaluation has continued up to 7 months postoperatively. The patient remains free of lameness or discomfort, with stable limb function confirmed during routine recheck visits for unrelated medical care.

## Discussion

Surgical correction of CrCL rupture in dogs has evolved significantly over the past decades, with techniques such as TPLO gaining widespread use in medium and large breeds due to their ability to neutralize cranial tibial thrust (3). However, applying these methods to toy-breed dogs is more complicated than simply scaling them down (4, 5).

Patients weighing less than 3 kg are particularly vulnerable to implant-related complications, including screw loosening, cortical fractures, and delayed healing. The reduced cortical thickness and bone volume inherent to small dogs limits the applicability of hardware-intensive procedures (5, 6). Furthermore, TPLO and similar osteotomy techniques primarily address sagittal plane instability and are less effective in correcting rotational laxity, which may be of greater functional relevance in smaller breeds with lower musculoskeletal demands (7).

In this context, combined stabilization strategies integrating intra-articular and extracapsular elements have gained interest. These approaches aim to replicate the anatomical orientation and function of the native CrCL without relying on osteotomy. Recent cadaveric studies have demonstrated that such hybrid methods can achieve improved control of both tibial translation and internal rotation (11). Consistent with these findings, recent biomechanical evidence supports that combined intra- and extracapsular stabilization offers superior control of both internal rotation and cranial tibial translation compared to intracapsular reconstruction alone, without imposing excessive restriction on other joint motions. In an *ex vivo* study using a weight-bearing gait simulation, Sun et al. (11) demonstrated that the addition of an extracapsular component to an intracapsular graft effectively restored near-physiologic stifle kinematics, particularly improving rotational and translational stability throughout the stance phase. This suggests that such hybrid constructs may better mimic native ligament biomechanics and reduce early postoperative graft elongation risk.

Comparative evidence from other species provides important biomechanical context for interpreting this single-case outcome. In bovine stifle models, both autograft and synthetic ligament reconstructions have been investigated and shown to restore joint stability under experimental conditions. Crawford (13) demonstrated functional recovery following intra-articular replacement of the transected CrCL in Holstein heifers using an autogenous fascial graft, while Constant et al. (14) biomechanically evaluated a braided superelastic nitinol bone-to-bone prosthesis that effectively reduced tibial translation after CrCL transection. Although these studies were performed independently, they collectively demonstrate that both biologic and synthetic ligament reconstructions can achieve functional stabilization within the same species. This interspecies evidence supports the mechanical plausibility of the surgical concept described in the present canine case and strengthens its scientific basis despite the inherent limitation of a single clinical observation.

Compared with conventional extracapsular fixation relying on fabellar anchorage, the present hybrid configuration provides more consistent cortical purchase through bone tunnels, reducing the risk of anchor pull-out and suture creep commonly reported in small-breed dogs. By distributing tensile forces between intraosseous and extracapsular components, the construct minimizes localized stress while maintaining physiologic joint motion. We did not employ fabellar anchorage in this case because fixation around the lateral fabella can be susceptible to failure modes such as suture elongation, suture rupture, or pull-out from the anchorage site—mechanisms documented in both clinical series and biomechanical studies. Moreover, anchor pull-out strength is strongly influenced by local cortical thickness and bone quality; in small and toy-breed dogs, the fabella and adjacent femoral cortex may provide insufficient purchase for secure fixation, increasing the risk of early failure (15). For these reasons, and given the patient's small bone geometry, we selected a hybrid intra-/extracapsular approach that avoid sole reliance on fabellar fixation while offering improved resistance to both translational and rotational instability (16).

Compared with osteotomy-based procedures such as TPLO or TTA, which typically require 8–12 weeks for complete osseous healing and restoration of full weight-bearing function, the hybrid intra-/extracapsular technique achieved complete functional recovery within 4 weeks and maintained stable limb function for 7 months without recurrence. This shorter rehabilitation period likely reflects preservation of native bone architecture and elimination of osteotomy-related complications such as tibial tuberosity fracture, delayed union, or implant irritation. Furthermore, the absence of hardware and minimal periosteal disruption may contribute to earlier proprioceptive recovery and reduced postoperative discomfort.

The management of CrCL rupture in toy-breed dogs presents a unique surgical challenge, particularly in patients with preexisting comorbidities or altered tibial anatomy. This case illustrates that a combined intra- and extracapsular stabilization strategy can offer a mechanically stable outcome without the risks associated with osteotomy-based methods.

The approach described here avoided the use of implants and preserved native bone architecture, while still addressing both translational and rotational instability. Early functional recovery was achieved, and no complications were observed during the postoperative period.

While limited by the single-subject nature of the report, this experience supports the exploration of alternative stabilization methods in high-risk or size-constrained canine patients. Broader application of such techniques should be guided by ongoing clinical validation through a prospective case series and long-term outcome monitoring across diverse patient populations, potentially supplemented by *ex vivo* biomechanical evaluation to objectively assess stability. Recent cadaveric and e*x vivo* biomechanical investigations have provided supportive evidence for this approach. Sun et al. (11) demonstrated that combined intra- and extracapsular constructs restored near-physiologic stifle kinematics under cyclic loading, confirming their ability to control both tibial translation and internal rotation. Similarly, Goin et al. (12) reported high fatigue resistance and fixation stability of synthetic intra-articular ligament systems in canine cadaver models. These findings objectively substantiate the mechanical validity of the presented technique and support its consideration as a feasible non-osteotomy alternative pending further clinical accumulation. This information may further assist clinicians and academic readers in making evidence-based interpretations, thereby providing reliable information for both clinical and scientific audiences.

## Data Availability

No dataset is publicly available due to the single case nature of the study. Requests to access the datasets should be directed to Sung Su Park, slugger98@hanmail.net.

## References

[1] Hayashi K Manley PA Muir P. Cranial cruciate ligament pathophysiology in dogs with cruciate disease: a review. J Am Anim Hosp Assoc. (2004) 40:385–390. doi: 10.5326/0400385 15347618

[2] Taylor-Brown FE Meeson RL Brodbelt DC Church DB McGreevy PD Thomson PC . Epidemiology of cranial cruciate ligament disease diagnosis in dogs attending primary-care veterinary practices in England. Vet Surg. (2015) 44:777–83. doi: 10.1111/vsu.12349 26118493

[3] Duerr FM Duncan CG Savicky RS Park RD Egger EL Palmer RH. Comparison of surgical treatment options for cranial cruciate ligament disease in large-breed dogs with excessive tibial plateau angle. Vet Surg. (2008) 37:49–62. doi: 10.1111/j.1532-950X.2007.00348.x 18199057

[4] Cosenza G Reif U Martini FM. Tibial plateau levelling osteotomy in 69 small breed dogs using conically coupled 19/25 mm locking plates: a clinical and radiographic retrospective assessment. Vet Comp Orthop Traumatol. (2015) 28:347–54. doi: 10.3415/VCOT-14-09-0135 26037208

[5] Marin K Unis MD Horgan JE Roush JK. Risk factors for short-term postoperative complications in dogs weighing < 15 kg after TPLO. PLoS ONE. (2021) 16:e0247555. doi: 10.1371/journal.pone.0247555 33630887 PMC7906318

[6] Fitzpatrick N Solano MA. Predictive variables for complications after TPLO with stifle inspection by arthrotomy in 1,000 consecutive dogs. Vet Surg. (2010) 39:460–74. doi: 10.1111/j.1532-950X.2010.00663.x 20345526

[7] Kim SE Lewis DD Pozzi A. Effect of tibial plateau leveling osteotomy on femorotibial subluxation: in vivo analysis during standing. Vet Surg. (2012) 41:465–70. doi: 10.1111/j.1532-950X.2012.00973.x 22380922

[8] Schuenemann R Kaczmarek J. Tibial plateau leveling osteotomy in small and large breed dogs: a comparative retrospective study of complications and outcomes. Tierarztl Prax Ausg K Kleintiere Heimtiere. (2023) 51:6–14. doi: 10.1055/a-1990-0597 36917988

[9] Bergh MS Rajala-Schultz P Johnson KA. Risk factors for tibial tuberosity fracture after tibial plateau leveling osteotomy in dogs. Vet Surg. (2008) 37:374–82. doi: 10.1111/j.1532-950X.2008.00391.x 18564262

[10] Muroi N Shimada M Murakami S Akagi H Kanno N Suzuki S . A retrospective study of postoperative development of implant-induced osteoporosis in radial-ulnar fractures in toy breed dogs treated with plate fixation. Vet Comp Orthop Traumatol. (2021) 34:375–85. doi: 10.1055/s-0041-1731810 34344053

[11] Sun CY Lin CC Wu CH. Ex vivo biomechanical investigations of combined extra- and intracapsular stabilization in canines with cranial cruciate ligament deficiency. Front Vet Sci. (2024) 11:1336797. doi: 10.3389/fvets.2024.1336797 38933704 PMC11199530

[12] Goin B Buttin P Lafon Y Massenzio M Viguier E Cachon T. Biomechanical cyclic loading test of a synthetic ligament fixation system used for intra-articular stabilization of deficient canine stifles. Open Vet J. (2022) 12:341–50. doi: 10.5455/OVJ.2022.v12.i3.6 35821774 PMC9270941

[13] Crawford WH. Intra-articular replacement of bovine cranial cruciate ligaments with an autogenous fascial graft. Vet Surg. (1990) 19:380–8. doi: 10.1111/j.1532-950X.1990.tb01213.x 2219676

[14] Constant C Braïlovski V Wagnac É Petit Y Desrochers A Nichols S. Biomechanical evaluation of bovine stifles stabilized with an innovative braided superelastic nitinol prosthesis after transection of the cranial cruciate ligament. Vet Surg. (2021) 50:1398–408. doi: 10.1111/vsu.13715 34435675

[15] Tonks CA Lewis DD Pozzi A. A review of extra-articular prosthetic stabilization of the cranial cruciate ligament-deficient stifle. Vet Comp Orthop Traumatol. (2011) 24:167–77. doi: 10.3415/VCOT-10-06-0084 21373714

[16] Brioschi V Arthurs GI. Cranial cruciate ligament rupture in small dogs (< 15 kg): narrative literature review. J Small Anim Pract. (2021) 62:1037–50. doi: 10.1111/jsap.13404 34269419

